# HPV vaccine stimulates cytotoxic activity of killer dendritic cells and natural killer cells against HPV-positive tumour cells

**DOI:** 10.1111/jcmm.12284

**Published:** 2014-06-30

**Authors:** Johan M J Van den Bergh, Khadija Guerti, Yannick Willemen, Eva Lion, Nathalie Cools, Herman Goossens, Alex Vorsters, Viggo F I Van Tendeloo, Sébastien Anguille, Pierre Van Damme, Evelien L J M Smits

**Affiliations:** a Laboratory of Experimental Hematology, Vaccine and Infectious Disease Institute (VAXINFECTIO), Faculty of Medicine & Health Sciences, University of AntwerpAntwerp, Belgium; b Division of Immunology, Department of Laboratory Medicine, Antwerp University HospitalEdegem, Belgium; c Laboratory of Medical Microbiology, Vaccine and Infectious Disease Institute (VAXINFECTIO), Faculty of Medicine & Health Sciences, University of AntwerpAntwerp, Belgium; d Centre for the Evaluation of Vaccination (CEV), Vaccine and Infectious Disease Institute (VAXINFECTIO), Faculty of Medicine & Health Sciences, University of AntwerpAntwerp, Belgium; e Center for Oncological Research Antwerp, Faculty of Medicine & Health Sciences, University of AntwerpAntwerp, Belgium

**Keywords:** human papillomavirus vaccine, dendritic cells, natural killer cells, virus-like particles, cytotoxicity

## Abstract

*Cervarix*™ is approved as a preventive vaccine against infection with the human papillomavirus (HPV) strains 16 and 18, which are causally related to the development of cervical cancer. We are the first to investigate *in vitro* the effects of this HPV vaccine on interleukin (IL)-15 dendritic cells (DC) as proxy of a naturally occurring subset of blood DC, and natural killer (NK) cells, two innate immune cell types that play an important role in antitumour immunity. Our results show that exposure of IL-15 DC to the HPV vaccine results in increased expression of phenotypic maturation markers, pro-inflammatory cytokine production and cytotoxic activity against HPV-positive tumour cells. These effects are mediated by the vaccine adjuvant, partly through Toll-like receptor 4 activation. Next, we demonstrate that vaccine-exposed IL-15 DC in turn induce phenotypic activation of NK cells, resulting in a synergistic cytotoxic action against HPV-infected tumour cells. Our study thus identifies a novel mode of action of the HPV vaccine in boosting innate immunity, including killing of HPV-infected cells by DC and NK cells.

## Introduction

Genital infection with oncogenic human papillomavirus (HPV) strains, such as HPV16 and HPV18, is the leading cause of cervical cancer. *Cervarix*™ is one of the two HPV vaccines that have been licensed and marketed in Europe and the United States for cervical cancer prevention [1,2]. This bivalent HPV vaccine contains virus-like particles (VLP) of the L1 protein of HPV16 and HPV18 combined with Adjuvant System 04 (AS04). The latter is composed of 3-O-desacyl-4′-monophosphoryl lipid A (MPL) adsorbed onto aluminium salt [3–5]. In turn, MPL is a detoxified derivative of the lipopolysaccharide (LPS) isolated from the Gram-negative bacterium *Salmonella minnesota* R595 strain [3], and is described to be a Toll-like receptor (TLR)4 agonist [6,7].

Until now, studies investigating the immunological mechanism of action of the HPV vaccine have dealt with B cells, T cells and monocyte-derived dendritic cells (DC) [3,8–11]. So far, these studies have revealed that the HPV vaccine works (*i*) by triggering a humoural immune response consisting of viral antigen-specific IgG antibodies and virus-neutralizing antibodies [9–11], (*ii*) by inducing a cellular immune response characterized by proliferation and activation of viral antigen-specific CD4^+^ T cells [8,10], and (*iii*) by activating DC to enhance adaptive immunity [3,8]. The latter results were obtained by using *in vitro*-generated interleukin (IL)-4-conditioned monocyte-derived DC (IL-4 DC). In recent years, the naturally existing CD56^+^CD7^−^CD11c^+^BDCA1^+^ blood DC subset has gained interest because of its potential direct cytotoxic activity [12,13]. Detailed functional studies, however, are complicated by the low frequencies of DC with this specific phenotype *in vivo* [13]. We and others have developed a novel *in vitro* DC culture system that allows for the generation of CD56^+^ DC (hereafter referred to as IL-15 DC) [14], representing the *in vitro* correlate of the naturally existing CD56^+^CD7^−^CD11c^+^BDCA1^+^ myeloid blood DC subset. A growing body of evidence has now accumulated to show that human DC, including IL-15 DC [15,16], can exert direct cytotoxic effects against cancer cells [17,18], which can be triggered by various immunostimulatory cytokines and TLR stimuli. Whether or not HPV vaccines can unlock the intrinsic cytotoxic effector function of IL-15 DC against HPV-positive tumour cells remains to be investigated.

In addition to DC, natural killer (NK) cells – the prime effectors of the innate immune system – play a key role in immune protection against HPV and cervical cancer [19,20]. Similar to what is observed in other malignancies [21], quantitative and qualitative abnormalities in the NK-cell compartment are frequently observed in cervical cancer or its precursor lesions. For example, the local NK-cell infiltrate in HPV-infected preneoplastic and neoplastic lesions of the cervix often displays a decreased expression of activating NK-cell receptors (*e.g*. NKp30, NKp46 and NKG2D) and a diminished cytotoxic activity [22]. These abnormalities have also been implicated in cervical cancer progression, further underscoring the importance of NK cells in immune protection against HPV and cervical cancer [22–24].

Until now, the effects of the HPV vaccine on human IL-15 DC and the subsequent interaction of these DC with NK cells have not been investigated. In this study, we assess the effects of the HPV vaccine and its individual components on the phenotype and function of IL-15 DC and DC-stimulated NK cells with specific attention for the cytotoxic activity of both cell types against HPV-positive tumour cells.

## Material and methods

### Ethics statement and cell material

This study was approved by the Ethics Committee of the University of Antwerp (Antwerp, Belgium) under the reference number 11/47/366. All experiments were performed by using blood samples from anonymous donors provided by the Antwerp branch of the Red Cross Blood Transfusion Center (Edegem, Belgium). The human HPV16^+^ CaSki cell line (cervical epidermoid carcinoma) and the human HPV18^+^ HeLa cell line (cervical adenocarcinoma) were obtained from the American Type Culture Collection (Rockville, MD, USA) and maintained in Roswell Park Memorial Institute 1640 (RPMI; Invitrogen, Merelbeke, Belgium) supplemented with 10% foetal bovine serum (FBS; Invitrogen). The human HPV^−^ K562 cell line (bone marrow chronic myelogenous leukaemia) was obtained from the Deutsche Sammlung von Mikroorganismen und ZellKulturen (Braunschweig, Germany) and maintained in Iscove's Modified Dulbecco's Medium (IMDM; Invitrogen) supplemented with 10% FBS. All cell lines were maintained in logarithmic growth phase at 37°C in a humidified atmosphere supplemented with 5% CO_2_.

### IL-15 dendritic cells

CD14^+^ monocytes were isolated from peripheral blood mononuclear cells obtained after Ficoll-Paque Plus gradient separation (GE Healthcare, Uppsala, Sweden) by positive selection by using magnetic CD14 microbeads (Miltenyi Biotec, Utrecht, The Netherlands) according to the manufacturer's instructions. Monocytes were used to generate immature DC (iDC) as described previously with minor modifications [14]. Briefly, CD14^+^ monocytes were seeded in 75 cm^2^ polystyrene tissue culture flasks (Sarstedt Inc., Newton, NC, USA) at a concentration of 1.0–1.4 × 10^6^ cells/ml in RPMI 1640 culture medium supplemented with 2.5% heat-inactivated human AB serum (Invitrogen), 800 IU/ml granulocyte macrophage colony-stimulating factor (GM-CSF; Gentaur, Brussels, Belgium) and 200 ng/ml IL-15 (Immunotools, Friesoythe, Germany) and incubated at 37°C, 5% CO_2_.

After 48 hrs of differentiation, iDC were stimulated with (*i*) 0.5 μg/ml bivalent HPV vaccine *Cervarix*™(GlaxoSmithKline, GSK, Rixensart, Belgium) per 10^6^ cells (*i.e*. Cer-DC), (*ii*) 1 μg/ml purified HPV16 and/or HPV18 L1 VLP without adjuvant (kindly provided by GSK; *i.e*. VLP-DC), (*iii*) 1 μg/ml LPS (*i.e*. LPS-DC; Invivogen, San Diego, CA, USA) or (iv) left unstimulated (*i.e*. iDC). In specific experiments, 1 μg/ml of the TLR4 signalling inhibitor CLI-095 (Invivogen) was added to the culture medium of iDC 6 hrs before stimulation with LPS or the HPV vaccine to determine the role of TLR4 in the HPV vaccine's effect. After 18 hrs of stimulation at 37°C and 5% CO_2,_ cells were harvested and spun down. Culture supernatants were collected and stored at −20°C until further analysis (cytokine secretion profile). Cells were either stained for phenotypic analysis or resuspended in fresh medium for functional assays.

### Natural killer cells

Untouched resting CD3^−^CD56^+^ NK cells were isolated from the CD14^−^ peripheral blood lymphocyte fraction by using the human negative selection NK-cell isolation kit (Miltenyi Biotec) according to the manufacturer's instructions. NK-cell purity and viability was at least 95% as determined on a FACScan flow cytometer (BD, Erembodegem, Belgium) following staining with fluorescein isothiocyanate (FITC)-conjugated anti-CD3 and phycoerythrin (PE)-conjugated anti-CD56 monoclonal antibodies (BD), and propidium iodide (PI; Invitrogen), respectively. Purified NK cells were cryopreserved at −80°C until further use in freezing medium consisting of FBS supplemented with 10% dimethyl sulfoxide (Sigma-Aldrich, Diegem, Belgium).

### Membrane immunophenotyping

Dendritic cells were membrane stained with FITC-, PE-, allophycocyanin (APC)-, Pacific Blue- or V450-conjugated monoclonal antibodies (BD) against CD14, CD80, CD83, CD86, CD209, HLA-DR and IL-15. Thawed NK cells were cocultured at a 1:1 ratio with autologous DC in IMDM + 10% FBS and membrane stained with anti-NKp46-APC (BD), anti-NKG2D-PE (R&D, Abingdon, Oxon, UK) and anti-CD69-FITC (BD) monoclonal antibodies 4 hrs, 24 hrs, 42 hrs and 72 hrs after initiation of cultures. Corresponding isotype staining was performed as negative control. All samples were measured on a FACSAria II flow cytometer (BD).

### Cytokine secretion assay

Secretion of IL-1β, IL-2, IL-4, IL-5, IL-10, IL-12p70, IL-13, interferon (IFN)-γ and tumour necrosis factor (TNF)-α by DC was determined in supernatant by using a human T helper (Th)1/Th2 10–plex kit for electrochemiluminescent detection [Meso Scale Discovery (MSD), Rockville, MD, USA] and performed according to the manufacturer's protocol. Data were analysed on a SECTOR instrument (MSD) by using MSD's Discovery Workbench software.

### Cytotoxicity assay

The killing capacity of DC and NK cells against tumour cells was determined by using a flow cytometry-based protocol as described previously with minor modifications [15,25]. Briefly, tumour cells were labelled prior to coculture with PKH67 Green Fluorescent Cell Linker dye (Sigma-Aldrich) according to the manufacturer's protocol. PKH67^+^ tumour cells were cocultured with DC and/or NK cells for 4 hrs at different ratios followed by staining with annexin V-APC (BD) and PI. Tumour cells cultured without effector cells, *i.e*. DC or NK cells, served as controls. All samples were measured on a FACSAria II flow cytometer. Cytotoxicity was calculated based on the viability (annexin V^−^/PI^−^) of PKH67^+^ tumour cells by using the following equation:


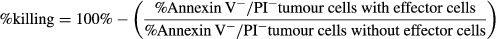


### Cytotoxic phenotyping

The cytotoxic phenotype of DC was determined by flow cytometry following staining for the most common cytotoxicity markers. DC were membrane stained for CD107a, CD178 (Fas ligand), and TNF-related apoptosis-inducing ligand (TRAIL) and stained intracellular for perforin and granzyme B by using FITC-, PE- and APC-conjugated monoclonal antibodies (BD). Corresponding isotype staining was performed as negative control. For intracellular staining, 10 μg/ml Brefeldin A (Invitrogen) was added to DC cultures 3 hrs prior to harvesting. After harvesting, cells were treated sequentially with FACS lysing and permeabilizing solution 2 (BD), and incubated with antibodies against perforin and granzyme B for 4 hrs at 4°C. All samples were measured on a FACSAria II flow cytometer.

### Statistical analysis

Flow cytometry data were analysed by using FlowJo version 10.0.6 (Treestar, Ashland, OR, USA). GraphPad Prism 5 software (GraphPad, San Diego, CA, USA) was used for graphing and statistical calculations. Statistical analysis was performed with the Wilcoxon matched-pairs test or repeated-measures one-way anova with Bonferroni post-hoc test, where appropriate. Results were considered statistically significant when *P* < 0.05.

## Results

### The HPV vaccine activates IL-15 DC

**Fig. 1:**
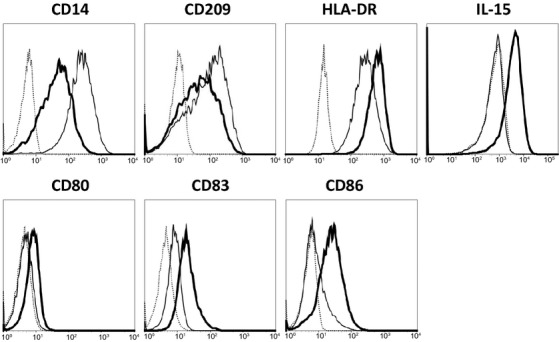
HPV vaccine induces phenotypic maturation of IL-15 DC. Histogram overlays show the surface expression of CD14, CD209, HLA-DR, IL-15, CD80, CD83 and CD86 of 18 hrs HPV vaccine-stimulated IL-15 DC (Cer-DC, bold-lined histogram) unstimulated iDC (thin-lined histogram) and isotype-matched controls (dashed-lined histogram). Histogram overlays are shown for one representative donor out of three independent donors.

As shown in Figure 1, stimulation of IL-15 DC with the HPV vaccine (*i.e*. Cer-DC) resulted in marked phenotypic activation. This was demonstrated by down-regulation of the immature DC markers CD14 and CD209 and by up-regulation of HLA-DR, the co-stimulatory molecules CD80/CD86 and the phenotypic maturation marker CD83. Interestingly, Cer-DC, but not unstimulated DC (*i.e*. iDC), also showed surface expression of the immunostimulatory cytokine IL-15.

A multiplexed electrochemiluminescent assay was performed to further evaluate the effect of the HPV vaccine on the cytokine production profile of IL-15 DC (Table 1). Upon HPV vaccine stimulation, the levels of the typical Th_1_-polarizing cytokine IFN-γ and of the pro-inflammatory cytokines IL-1β and TNF-α were significantly elevated (*P* < 0.01, *P* < 0.001, *P* < 0.001, respectively; *n* = 3), while typical Th_2_-polarizing cytokines IL-4, IL-5, IL-10 and IL-13 were undetectable.

**Table 1:** Effect of the HPV vaccine and its L1 VLP on the cytokine secretion profile of IL-15 DC

	No stimulus (pg/ml)	HPV16 VLP (pg/ml)	HPV18 VLP (pg/ml)	HPV16 VLP + HPV18 VLP (pg/ml)	HPV vaccine (pg/ml)
Typical Th1-polarizing					
IFN-γ	2 ± 2	4 ± 6	3 ± 5	0 ± 0	1075 ± 486**
IL-12p70	0 ± 0	0 ± 0	0 ± 0	0 ± 0	1 ± 0
IL-2	0 ± 0	1 ± 0	0 ± 0	0 ± 0	2 ± 0
Typical Th2-polarizing					
IL-4	0 ± 0	0 ± 0	0 ± 0	0 ± 0	0 ± 0
IL-5	0 ± 0	0 ± 0	0 ± 0	0 ± 0	0 ± 0
IL-10	0 ± 0	0 ± 0	0 ± 0	1 ± 1	2 ± 2
IL-13	0 ± 0	0 ± 0	0 ± 1	0 ± 0	2 ± 2
Pro-inflammatory					
IL-1β	9 ± 4	5 ± 3	13 ± 7	4 ± 3	440 ± 109***
TNF-α	7 ± 1	7 ± 4	6 ± 2	9 ± 2	3109 ± 407***

HPV, human papillomavirus; IFN, interferon; IL, interleukin; Th, T helper; TNF, tumor-necrosis factor; VLP, virus-like particle.

**P < 0.01, ***P < 0.001 (compared between HPV vaccine-stimulation and no stimulus; n = 3).

### The stimulatory effects of the HPV vaccine on IL-15 DC are partly mediated *via* TLR4

**Fig. 2:**
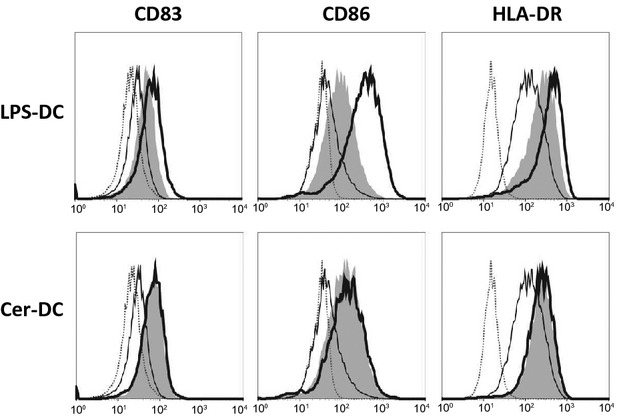
The effect of TLR4 blocking on HPV vaccine-induced DC maturation. Surface expression of CD83, CD86 and HLA-DR on DC after 18 hrs stimulation with LPS (LPS-DC, upper row) or the HPV vaccine (Cer-DC, lower row) in the absence (bold-lined histogram) or presence (grey filled histogram) of the TLR4 inhibitor CLI095 was compared with unstimulated iDC (thin-lined histogram) and isotype controls (dashed-lined histogram). CLI095 was added 6 hrs before LPS or HPV vaccine stimulation. Histogram overlays are shown for one representative donor out of three independent donors. Abbreviations: CLI095, TLR4 inhibitor; LPS, lipopolysaccharide.

We next aimed to dissect which component of the HPV vaccine was responsible for the above-observed stimulatory effects on IL-15 DC. Exposure of the DC to purified L1 HPV VLP without the AS04 adjuvant (*i.e*. VLP-DC) did not result in phenotypic activation (data not shown), nor did it affect the cytokine secretion profile of the DC (Table 1). To examine the possible role of the AS04 adjuvant, which contains the TLR4 ligand MPL, blocking experiments by using the TLR4 signalling inhibitor CLI095 were performed. We first determined a working concentration of the TLR4 inhibitor by exposing DC to the TLR4 agonist LPS (*i.e*. LPS-DC). As shown in Figure 2, at a concentration of 1 μg/ml CLI095, the LPS-mediated up-regulation of CD83, CD86 and HLA-DR on IL-15 DC could be clearly inhibited (delta MFI ± SEM = 11.9 ± 4.4 for CD83; delta MFI ± SEM = 201.4 ± 7.3 for CD86; delta MFI ± SEM = 284 ± 185 for HLA-DR). However, using the same concentration of the inhibitor, the HPV vaccine-induced up-regulation of these phenotypic markers was not affected (delta MFI ± SEM = 0.25 ± 0.9 for CD83; delta MFI ± SEM = 13.3 ± 0.3 for CD86; delta MFI ± SEM = 51.5 ± 57.5 for HLA-DR; Fig. 2).

Interestingly, while blocking the TLR4 pathway did not affect the phenotype of Cer-DC, cytokine production by Cer-DC was almost completely abolished by inhibiting TLR4 signalling (Table 2). These findings suggest that at least part of the stimulatory effects of the HPV vaccine on IL-15 DC is mediated through TLR4 signalling.

**Table 2:** Effect of TLR4 blocking on cytokine secretion by HPV vaccine-stimulated IL-15 DC

	No stimulus (pg/ml)	LPS		HPV vaccine	
			
		No CLI095 (pg/ml)	With CLI095 (pg/ml)	No CLI095 (pg/ml)	With CLI095 (pg/ml)
Typical Th1-polarizing					
IFN-γ	17 ± 6	2735 ± 1121	38 ± 15**	781 ± 368	41 ± 15*
IL-12p70	0 ± 0	28 ± 8	0 ± 0	0 ± 0	0 ± 0
IL-2	1 ± 1	4 ± 1	1 ± 1	2 ± 1	1 ± 0
Typical Th2-polarizing					
IL-4	1 ± 0	3 ± 1	1 ± 0	1 ± 0	0 ± 0
IL-5	0 ± 0	5 ± 1	1 ± 0	2 ± 1	1 ± 0
IL-10	2 ± 1	659 ± 614	33 ± 29	125 ± 115	1 ± 0
IL-13	2 ± 2	16 ± 4	2 ± 2	7 ± 2	0 ± 0
Pro-inflammatory					
IL-1β	61 ± 46	1452 ± 84	170 ± 38**	697 ± 322	109 ± 15*
TNF-α	37 ± 12	8443 ± 226	345 ± 273***	3430 ± 1699	447 ± 273*

CLI095, TLR4 inhibitor; HPV, human papillomavirus; IFN, interferon; IL, interleukin; Th, T helper; TLR4, Toll-like receptor 4; TNF, tumor-necrosis factor; LPS, lipopolysaccharide.

*P < 0.05, **P < 0.01, ***P < 0.001 (compared between CLI095 and no CLI095 added; n = 2).

### Cer-DC are cytotoxic against HPV16^+^ and HPV 18^+^ cervical cancer cells

**Fig. 3:**
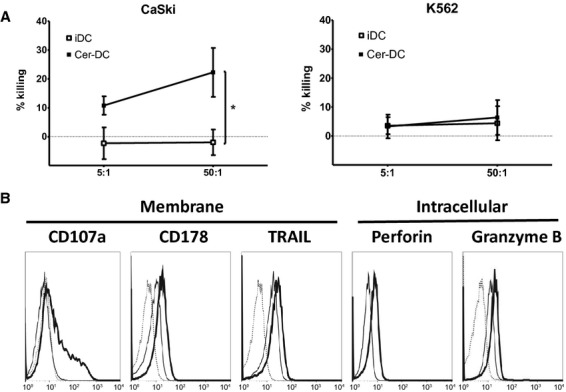
HPV vaccine stimulates functional and phenotypic cytotoxic properties on IL-15 DC. (**A**) The cytotoxicity capacity of iDC (open squares) and HPV vaccine-stimulated DC (Cer-DC, filled squares) against the HPV16^+^ cervical cancer cell line CaSki and the HPV^−^ CML cell line K562 was assessed at effector:target ratios of 5:1 and 50:1 in a 4 hrs flow cytometric cytotoxicity assay and represented as the mean (±SEM) killing percentage of target cells based on annexin V/PI staining (*n* = 3; *P < 0.05). (**B**) Histogram overlays of one representative donor out of three independent donors represent membrane (CD107a, CD178, TRAIL) and intracellular (perforin, granzyme B) expression of cytotoxic markers on 18 hrs HPV vaccine-stimulated DC (Cer-DC, bold-lined histogram), iDC (thin-lined histogram) and corresponding isotype controls (dashed-lined histogram). Abbreviations: PI, propidium iodide; SEM, standard error of mean; TRAIL, TNF-related apoptosis-inducing ligand.

The direct cytotoxicity of HPV vaccine-stimulated IL-15 DC (*i.e*. Cer-DC) was examined in a flow cytometric lysis assay by co-culturing DC with the HPV16^+^ cervical cancer cell line CaSki, the HPV18^+^ cervical cell line HeLa or the HPV^−^ CML cell line K562 for 4 hrs at an effector:target (E:T) ratio of 5:1 and 50:1. As shown in Figure 3A, Cer-DC exhibited significant cytotoxicity against CaSki at both the 5:1 (10.8 ± 1.8%) and 50:1 (22.3 ± 4.9%) ratios, whereas iDC exerted no demonstrable lytic activity (*P* < 0.05; Fig. 3A). Similar cytotoxic activity of Cer-DC was observed against HeLa cells (Cer-DC compared to iDC; delta% killing ± SEM = 3.5 ± 0.2 at 5:1 ratio; delta% killing ± SEM = 18.2 ± 1.8 at 50:1 ratio). Strikingly, neither iDC nor Cer-DC exerted a demonstrable lytic activity against the HPV^−^ K562 both at the 5:1 (% killing ± SEM = 3.6 ± 2.9 for iDC;% killing ± SEM = 3.3 ± 4.1 for Cer-DC) and 50:1 ratio (% killing ± SEM = 4.4 ± 5.9 for iDC;% killing ± SEM = 6.4 ± 6.0 for Cer-DC; Fig. 3A).

From a mechanistic point of view, we found that Cer-DC, and to a lesser extent the non-cytotoxic iDC, expressed different cytotoxic effector molecules on their cell surface, such as CD178 (Fas ligand) and TRAIL (delta MFI ± SEM = 7.2 ± 0.1 for CD178; delta MFI ± SEM = 9.6 ± 1.8 for TRAIL; Fig. 3B). Furthermore, intracellular cytokine staining revealed that Cer-DC had increased levels of the granule-associated lytic molecules perforin and granzyme B as compared to iDC (delta MFI ± SEM = 1.4 ± 0.2 for perforin; delta MFI ± SEM = 0.3 ± 0.1). The expression of CD107a on the DC surface after stimulation with the HPV vaccine confirmed their degranulation potential (delta MFI ± SEM = 18.2 ± 12.3; Fig. 3B).

### Cer-DC phenotypically activate autologous NK cells

**Fig. 4:**
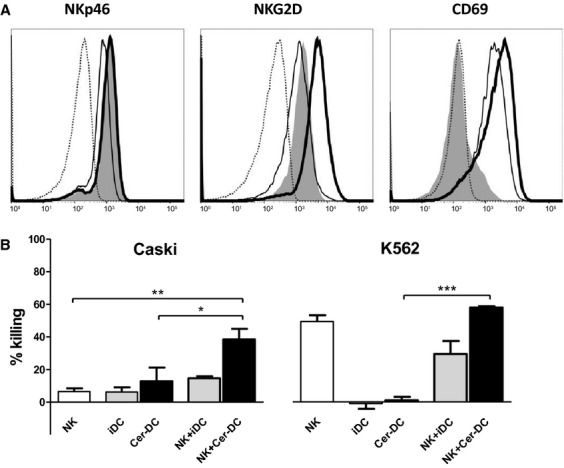
Phenotypic and functional NK-cell activation by HPV vaccine-stimulated DC. (**A**) Histogram overlays of one representative donor out of three independent donors represent NK-cell surface expression of NKp46, NKG2D and CD69 at their peak expression (42 hrs, 72 hrs and 42 hrs respectively) after coculture at a 1:1 ratio with autologous Cer-DC (bold-lined histogram) or iDC (thin-lined histogram), as compared with unstimulated NK cells (grey filled histogram) and corresponding isotype controls (dashed-lined histogram). (**B**) The mean killing percentage (±SEM) of HPV16^+^ CaSki and HPV^−^ K562 is shown for NK-cell/tumour cell (ratio 5:1, white bars), DC/tumour cell (ratio 5:1, iDC grey bars, Cer-DC black bars) and NK-cell/DC/tumour cell (ratio 5:5:1, NK-cell/iDC grey bars, NK-cell/Cer-DC black bars) co-cultures based on a 4 hrs flow cytometric cytotoxicity assay following 42 hrs NK-cell and/or DC co-cultures (*n* = 3). *P < 0.05, **P < 0.01, ***P < 0.001. Abbreviations: SEM, standard error of mean.

Natural killer cells were exposed to autologous iDC or Cer-DC for 4 hrs, 24 hrs, 42 hrs and 72 hrs, after which their surface expression of NKp46, NKG2D and CD69 was evaluated (Fig. 4A). Although Cer-DC did not further enhance the expression of the activating NK-cell receptor NKp46 (measured at the 42 hrs peak expression time-point), both the activating receptor NKG2D (72 hrs peak expression time-point) and the activation marker CD69 (42 hrs peak expression time-point) were found to be up-regulated on the NK-cell surface after stimulation with Cer-DC as compared to iDC (Fig. 4A).

### Cer-DC/NK-cell interaction synergistically increases cytotoxicity against HPV16^+^ and HPV18^+^ cervical cancer cells

In line with their ability to induce phenotypic activation of NK cells, Cer-DC were also found to interact with NK cells to enhance cytotoxicity against the HPV16^+^ CaSki and HPV18^+^ HeLa cervical cancer cell lines. As shown in Figure 4B, a significantly higher killing of CaSki cells was observed in the Cer-DC/NK-cell co-cultures (38.5 ± 6.5%), as compared to NK cells alone (6.4 ± 2.0%; *P* < 0.01) or Cer-DC alone (12.8 ± 8.4%; *P* < 0.05; Fig. 4B). Similar results were obtained with HeLa cells, with a significantly higher killing of HeLa cells in the Cer-DC/NK co-cultures (42.5 ± 15.8%), as compared to NK cells alone (7.4 ± 2.6%; *P* < 0.01) or Cer-DC alone (8.7 ± 1.4%; *P* < 0.01). For the HPV^−^ K562 cells, the percentage of killed cells was also significantly higher in the Cer-DC/NK-cell co-cultures (77.7 ± 1.8%), as compared with Cer-DC alone (1.3 ± 4.9%; *P* < 0.001), but comparable to NK cells alone (66.2 ± 9.0%; *P* > 0.05; Fig. 4B).

## Discussion

The *Cervarix*™ HPV vaccine, consisting of truncated HPV16 and HPV18 VLP in combination with the adjuvant AS04, has been developed to prevent cervical cancer caused by HPV infections [26]. It has been demonstrated that this HPV vaccine elicits antigen-specific and virus-neutralizing antibody responses and CD4^+^ T-cell responses [10,11,27]. Furthermore, it was shown that both the adjuvant AS04 (containing MPL) and MPL alone induce DC maturation, which further increases antigen-specific T-cell responses [3,8]. Until now, however, the effects of the HPV vaccine (or its components) on DC were investigated by using *in vitro*-generated IL-4-conditioned DC [3,8]. Here, we are the first to examine the effects of the vaccine on *in vitro*-generated IL-15-conditioned DC. These IL-15 DC have a unique phenotype, including the expression of CD56, which may be involved in the direct cytotoxic potential of these cells (so-called killer DC) [14]. It has only recently been appreciated that this cell surface marker is not specific for lymphoid cells (*e.g*. NK cells), but is also present on cells with myeloid-lineage orientation [12,13]. The latter can be discriminated from the lymphoid cells by their lack of CD7 expression and can be further subdivided into DC-like monocytic cells (CD14^+^) and blood myeloid DC (CD11c^+^ and BDCA1^+^). As the frequency of the naturally existing blood myeloid cells with the CD56^+^CD7^−^CD11c^+^BDCA1^+^ phenotype is low *in vivo* [13], we used an *in vitro-*related subset, *i.e*. IL-15 DC [14], to investigate the effects of the HPV vaccine.

With this study, we demonstrate that the complete HPV vaccine, but not the VLP alone, leads to a higher expression of the antigen-presenting molecule HLA-DR, the co-stimulatory molecules CD80/CD86 and the phenotypic maturation marker CD83 on IL-15 DC. Similar effects on IL-4-conditioned DC have been reported by other research groups by using full-length VLP of HPV16 and HPV18 [28,29] (as opposed to the truncated HPV VLP in *Cervarix*™) or the adjuvant AS04 [3]. In addition to phenotypic activation, we showed that Cer-DC, but not VLP-DC, produced high amounts of the pro-inflammatory cytokines IFN-γ, IL-1β, and TNF-α, but no detectable levels of Th2-polarizing cytokines. IFN-γ is an important signalling molecule in the differentiation of Th1 cells, which are highly favourable in the battle against infections and tumour cells [30]. Interleukin-12, another Th1-polarizing cytokine, on the other hand, was not detected after stimulation with the HPV vaccine. An explanation for this could be the absence of a second stimulatory signal, *in concreto* CD40-CD40L interaction, as we did not observe expression of CD40 on Cer-DC [14,31].

While others have described that HPV VLP alone can induce the activation of DC [28,29,32], we did not detect an effect of the adjuvant-free L1 HPV VLP on DC maturation, nor on cytokine secretion, not even with a doubled concentration. A possible reason for these contrasting observations might be the difference in DC that were used, because Langerhans cells (in contrast to conventional IL-4 DC) were found to be refractory to activation by VLP [33]. Like Langerhans cells, but unlike IL-4 DC, the IL-15 DC used in our study were shown to express langerin [14,34], which could explain why they were not activated by VLP alone. Therefore, HPV VLP were not included in functionality tests of either DC or NK cells.

Besides VLP, the HPV vaccine contains the TLR agonist MPL (in the adjuvant AS04), a detoxified derivative of LPS that maintains most of its immunostimulatory properties and operates as a potent TLR4 agonist [35]. Hence, we evaluated the dependence on TLR4 signalling by the adjuvant for vaccine-induced DC activation. We found that blocking TLR4 signalling with the inhibitor CLI095 almost completely abolished the HPV vaccine's effects on DC cytokine secretion. In contrast, we observed that the effects on the phenotype of DC were not affected by blocking TLR4. As MPL is also described as a possible TLR2 agonist, TLR2 signalling (in addition to TLR4 signalling) might be involved in the HPV vaccine's effects on DC activation [36].

In addition to the effects on maturation and cytokine secretion, we are the first to demonstrate that the HPV vaccine stimulates the intrinsic innate function of IL-15 DC, resulting in the killing of HPV16^+^ and HPV18^+^ cervical cancer cells, but not HPV^−^ K562 cells. This indicates that the presence of active virus is needed for DC to exert cytotoxic functions against tumour cells. Upon flow cytometric analysis of the most common membrane and intracellular cytotoxicity markers, we discovered that Cer-DC express CD107a, CD178 (Fas ligand), TRAIL, perforin and granzyme B. Cytotoxicity of Cer-DC against HPV^+^ tumour cells, in combination with the expression of cytotoxic markers on these DC, is in concordance with others who described that DC can be cytotoxic against tumour cells by means of TNF family molecules (TRAIL, CD178) expressed on the cell surface of activated DC [37]. As shown by our research group and others, perforin and/or granzyme B-producing DC are also capable of killing tumour cells [15,38,39], which favour the idea that the increased killing of CaSki tumour cells occurs *via* a perforin/granzyme B-dependent mechanism. In this context, increasing or unlocking the intrinsic killer function of myeloid blood DC could contribute to a strong immune response against HPV-infected cells.

As NK cells are the prime effector cells of the innate immune system and play a key role in the immune protection against HPV and cervical cancer, stimulating NK-cell functions could favour HPV immunity. We demonstrated that the HPV vaccine can indirectly promote NK-cell immunity *via* IL-15 DC, illustrated by phenotypic NK-cell activation and synergistically increased cytotoxicity against HPV16^+^ and HPV18^+^ cervical cancer cells by Cer-DC/NK-cell co-cultures. Our observations hereby support reports by others showing that IL-15- and TNF-α-producing DC are capable of inducing or increasing NK-cell function [40], because Cer-DC were found to produce both IL-15 (on the cell surface) and TNF-α. Moreover, IL-15 disappeared from the Cer-DC surface during coculture with autologous NK cells (data not shown), suggesting uptake of this cytokine by NK cells.

As the HPV vaccine is capable of unlocking the cytotoxic function of IL-15 DC against HPV^+^ tumour cells and as cytotoxicity is further increased after NK/DC coculture, the HPV vaccine might have therapeutic effects. The local NK-cell infiltrate in HPV-infected preneoplastic and neoplastic lesions of the cervix often displays a decreased expression of activating NK-cell receptors (*e.g*. NKp30, NKp46 and NKG2D) and a diminished cytotoxic activity [22]. These abnormalities are related to cervical cancer progression [22–24]. Thus, increasing the DC- and/or NK-mediated cytotoxicity by the HPV vaccine might improve the outcome of cervical cancer.

In conclusion, we are the first to show that stimulation of IL-15 DC (related to the naturally existing CD56^+^CD7^−^CD11c^+^BDCA1^+^ myeloid DC subset) with the HPV vaccine prompts phenotypic activation and cytokine secretion and that these effects are partially mediated through TLR4 signalling. Strikingly, HPV vaccine-stimulated IL-15 DC exert cytotoxic activity against HPV16^+^ and HPV18^+^ cervical cancer cells, which is synergistically increased when these DC are combined with autologous NK cells. Hence, our data identify a novel mode of action by which the HPV vaccine can protect against cervical cancer development.
